# Multiplex digital spatial profiling identifies subregion dependent targeted proteome changes across variants of dementia

**DOI:** 10.1038/s44400-025-00010-6

**Published:** 2025-06-03

**Authors:** MacKenzie L. Bolen, Kelly B. Menees, Marla Gearing, Jingjing Gong, Yuqi Ren, Andrea R. Merchak, Melissa E. Murray, Zachary T. McEachin, Malú Gámez Tansey

**Affiliations:** 1 Center for Translational Research in Neurodegenerative Disease, College of Medicine, University of Florida, Gainesville, FL USA; 2 Department of Neuroscience, College of Medicine, University of Florida, Gainesville, FL USA; 3 McKnight Brain Institute, University of Florida, Gainesville, FL USA; 4 Aligning Science Across Parkinson’s (ASAP) Collaborative Research Network, Chevy Chase, MD USA; 5 Department of Human Genetics, Emory University School of Medicine, Atlanta, GA USA; 6 Center for Neurodegenerative Disease, Emory University School of Medicine, Atlanta, GA USA; 7 NanoString Technologies Inc, Seattle, WA USA; 8 Department of Neuroscience, Mayo Clinic Florida, Jacksonville, FL USA; 9 Laboratory of Translational Cell Biology, Emory University School of Medicine, Atlanta, GA USA; 10 Department of Pathology & Laboratory Medicine, Emory University School of Medicine, Atlanta, GA USA; 11 Norman Fixel Institute for Neurological Diseases, University of Florida, Gainesville, FL USA

**Keywords:** Neurological disorders, Diagnostic markers, Imaging, Neuroscience, Molecular neuroscience

## Abstract

Frontotemporal lobar degeneration (FTLD) is the leading cause of dementia in patients under the age of 65. Even in a single anatomical region, there is variance within pathological protein deposition within the FTLD spectrum, which drives difficulty in post-mortem clinicopathological diagnoses. We spatially multiplexed the proteome geography at two levels of the cortex and the subcortical white matter in patients with various types of dementia (Alzheimer’s disease, C9orf72, MAPT also referred to as FTLD-tau, FTLD-TDP, FTLD-GRN; *n* = 6 per syndrome) and neurologically healthy controls (NHC). Layers II-V of the cortex from diseased individuals displayed the greatest protein dysregulation as compared to NHC. Traditional biomarkers of dementia, like phosphorylated tau proteins and Aβ42 displayed dysregulation, however, our data suggest spatial enrichment distinct to cortical sublayers. In conclusion, the specific localization of these protein deposits could be used to elucidate region-specific pathologic biomarkers unique to individual variants of dementia.

## Introduction

FTLD encapsulates a group of degenerative proteinopathies associated with the early breakdown of the frontal and temporal lobes of the brain and is the neuropathological diagnosis corresponding to clinical frontotemporal dementia (FTD)^1,2^. Early stages of disease are commonly characterized by language difficulties and behavioral abnormalities, while later stages of FTLD constitute a rapid cognitive decline paired with dementia^1,3^. There are three primary clinical groupings of FTD: the behavioral variant (bvFTD), progressive non-fluent aphasia, and movement semantic dementia^1,4^. However, even within these groupings, there is vast non-linear heterogeneity across disease pathology, etiology, and symptomology^5^, which has presented significant challenges to therapeutic research and pathological diagnosis within the FTLD spectrum.

Up to 40% of all FTLD cases are associated with various genetic variants^4,5^. The most common genetic variants associated with FTLD are linked to changes in the microtubule associated protein tau *(MAPT*), progranulin (*PGRN*), chromosome 9 open reading frame 72 (*C9ORF72*), and transactive DNA-binding protein gene (*TARDBP*) that expresses TDP-43(TAR DNA-binding protein 43)^6^.

Interestingly, FTD presentation and prognosis can overlap to some extent with Alzheimer’s disease (AD), Parkinson’s disease (PD), and amyotrophic lateral sclerosis (ALS)—making it difficult to distinguish FTD from AD, PD, or ALS until the pathological diagnosis can be determined^7–9^. Making the diagnosis even more complicated is comorbid disease onset; meaning a patient can clinically present with FTD but the pathological diagnosis at autopsy may include secondary pathology such as AD neuropathologic changes, Lewy bodies, or TDP-43 positive inclusions.

A newly emerging thread of investigation to better diagnose FTLD and related dementias postmortem has been region-specific analysis of protein deposition. FTLD appears to induce syndrome-specific protein deposition^10,11^; however, the distinct protein community(s) unique to each cortical subregion have yet to be characterized. The cortical layers each have unique cellular compositions, resulting in a diverse array of complex functions. With the understanding that the catalyst of FTLD can be patient specific and extremely variable, the current frontier of FTLD research has moved towards a more multiplexed approach to investigate the role laminar cortical pathology plays in the clinical syndrome and proteinopathies associated with FTLD and related dementias.

In this study, we aimed to identify differentially abundant proteins in a spatially resolved manner across several neurodegenerative diseases including AD and several FTLD subtypes. Here, we utilize NanoString GeoMx^TM^ DSP, a novel method that leverages the ability to spatially multiplex a community of protein biomarkers of interest. This technology provides a single system approach to investigating the region-specific pathologic proteomic contribution of specific disease states, which will lay the groundwork for future biomarker investigation and clarification of the FTLD disease spectrum.

## Results

### Cortical sublayers reveal disease-relevant shared and distinct protein expression

**Fig. 1:**
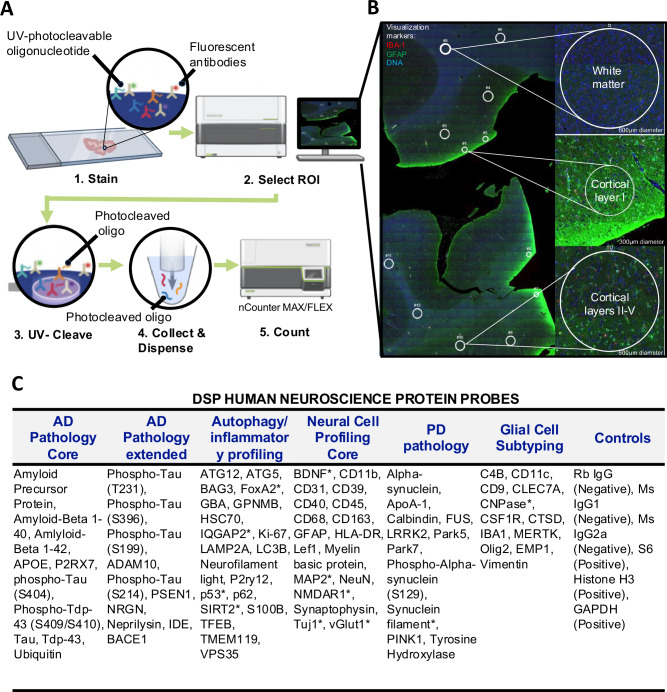
Digital spatial proteomic profiling (DSP) using immunofluorescence as an indicator of multiple proteomic targets on a single slide. **A** Working procedure of NanoString GeoMx Digital Spatial Profiling using nCounter MAX/FLEX. Paraformaldehyde-fixed cortical human tissue is incubated with 3 antibody visualization markers, followed by incubation with 75 DSP probes. After tissue is imaged, regions of interest (ROI) are digitally selected, ROIs are then subjected to ultraviolet (UV) light which results in release of UV-cleavable protein specific oligonucleotide barcodes from DSP probes. Cleaved protein unique barcodes are then collected and quantified. **B** Microphotograph of cortical tissue labeled with Iba1 (red), GFAP (green), and DNA (blue) visualization markers. Regions of interest (ROI) were manually drawn in approximately the same location within white matter, molecular layer (cortical layer I), and internal/granular layer (cortical layer IV) for every case. Four ROIs per subregion per patient (12 ROIs per tissue section) were collected and assessed as individual data points in all downstream analyses. Individual case demographics and cognitive status may be found in Supplementary Table 5. **C** DSP human Neuroscience protein probes, separated by disease or functional genre, a (*) indicates a protein that was not part of the NanoString Human Neuroscience panel but rather was chosen by our lab and spiked in.

**Fig. 2:**
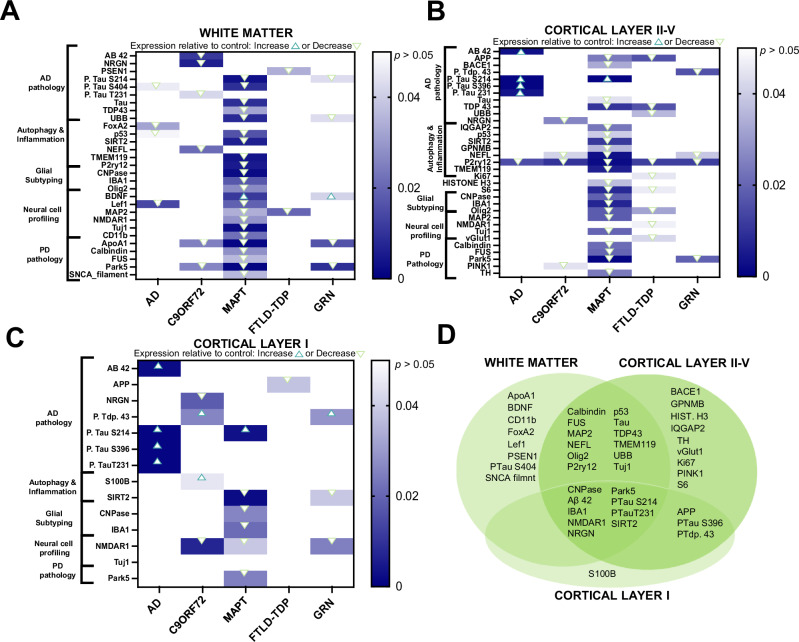
Cortical and subcortical laminar layers reveal significantly different disease specific proteomic expression relative to control. **A**–**C** Heatmaps of differentially expressed proteins when comparing all cortical subregions of interest across disease diagnosis. Linear mixed model regression analysis was used to identify significantly upregulated proteins in disease groups are indicated as any value above zero but less than *p* < 0.05. Significantly (*p* < 0.05) downregulated proteins are indicated by any value below zero. Individual significance and fold change values by disease within each subregion can be found in Supplementary Table 1-4. **D** Venn diagram depicting proteins distinct to cortical subregions within all disease groups that are significantly different than control. All counts were normalized to ROI area and internal controls.

By employing the NanoString GeoMx^TM^ DSP platform we assessed the regulation of 75 different proteins across 3 separate layers (molecular layer I, internal layers II-V, and white matter) of cortical tissue, in 5 different types of dementia (Fig. 1A–C). Individual cortical subregion region of interest (ROI) analysis of the cortex revealed differential disease associated protein regulation (Fig. 2). Differential expression was fit on a per protein basis using a linear mixed model. The model used the protein expression as the dependent variable and fit the cortical subregions and diseases as a fixed effect respectively to test for differences between groups. Patient was fit as a random effect. We grouped these protein communities by NanoString recommended panel association or by autophagy and/or inflammation associated proteins (Fig. 1C), the composition of proteins drastically changes within each community based off cortical subregion. We were able parse known disease relevant biomarkers and communities of implicated biomarkers that are subregion dependent. For example, amyloid beta 42 (Aβ42) and phosphorylated tau S214, S396 and T231 deposition only significantly increased in cortical layer I and cortical layers II-V of the cortical tissue from AD patients assessed (Fig. 2B, C) but not the subcortical white matter (Fig. 2A). Interestingly, there was a significant decrease in phosphorylated tau species in subcortical white matter tissue in the AD, C9ORF72, MAPT, and GRN pathology groups (Fig. 2A) but a significant increase of phosphorylated tau species in cortical layers II-V and cortical layer I of the cortex in AD and MAPT tissue, as compared to neurologically healthy control (NHC) (Fig. 2B, C). When comparing significantly different protein expression by disease groups versus NHC, no disease group showed significant overlap in similar proteins expressed across cortical subregion (Fig. 2)—further meaning each disease group revealed a relatively unique disease specific protein expression by cortical layer. We direct the reader to supplemental material for all individual protein count statistical differences and fold changes for white matter (Supplementary Table 1), cortical layers II-V (Supplementary Table 2), and cortical layer I (Supplementary Table 3).

### Spatial profiling of cortical white matter reveals significant protein downregulation in 5 variants of dementia across multiple cell types, compared to neurologically healthy control

Proteins downregulated across multiple diseases in subcortical white matter include Park-5 (C9ORF72, MAPT, GRN), ApoA1 (C9ORF72, MAPT, GRN), Map2 (MAPT and FTLD-TDP) phosphorylated tau S214 (MAPT and GRN), UBB (MAPT and GRN), p53 (AD and MAPT), and Lef1 (AD and MAPT) (Fig. 2A; Supplementary Table 1). The only upregulated protein across multiple diseases in the white matter was BDNF (MAPT and GRN) (Fig. 2A). The MAPT-positive cohort had the lowest expression of all significantly different proteins in the subcortical white matter as compared to AD, C9ORF72, FTLD-TDP, and GRN (Fig. 2A: Supplementary Table 1). Collectively, these data suggest that independent of disease, some protein deposition indicative of pathologic progression may be unique to specific cortical layers.

### Glial activation marker, P2ry12, is downregulated across all five variants of dementia in the internal cortical laminar layer

P2ry12 was significantly downregulated across all five disease groups, as compared to NHC in the internal subregion of the cortex (Fig. 2B; Supplementary Table 2). There was no other protein within our chosen panel that impacted all 5 disease groups within the same region. Proteins downregulated across multiple groups within the internal subregion of the cortex include APP (significant change identified in MAPT and FTLD-TDP), TDP-43 (significant change identified in MAPT and FTLD-TDP), NEFL (significant change identified in C9ORF72, MAPT and GRN), S6 (significant change identified in MAPT and FTLD-TDP), Olig2 (significant change identified in MAPT and FTLD-TDP) and Park-5 (significant change identified in MAPT and GRN) (Fig. 2B; Supplementary Table 2). The only protein upregulated across multiple diseases within the internal sublayer of the cortical tissue assessed was phosphorylated tau S214 (significant change identified in MAPT and GRN) (Fig. 2B; Supplementary Table 2). These data suggest that multiplexing P2ry12 with pathological indicators of disease, like TDP-43, NEF-L, and phosphorylated tau S214, could provide additional insight into parsing apart the discrepancies between dementia variant associated protein dysregulation and deposition.

### In cortical layer I, phosphorylated tau S214 and/or TDP-43 are upregulated in various forms of dementia but not all

Within cortical layer I of the cortex, NMDAR1 was downregulated in both C9ORF72 and GRN cortical tissue, but protein was upregulated in MAPT (Fig. 2C; Supplementary Table 3). The proteins upregulated across multiple neurodegenerative diseases within cortical layer I include phosphorylated TDP-43 (significant change identified in C9ORF72 and GRN) and phosphorylated tau S214 (significant change identified in AD and MAPT) (Fig. 2C; Supplementary Table 3). Phosphorylated TDP-43 and phosphorylated tau S214 are two of many pathologic lesions associated with FTLD^12^. Of interest is the region-specific upregulation of these markers increase that appears to be disease dependent within the FTLD spectrum. For example, phosphorylated tau S214 is upregulated in both cortical layers II-V and cortical layer I but down regulated in the white matter of cortical tissue positive for MAPT. These data reveal distinct spatial distribution of known pathology associated proteins in a disease dependent fashion.

### Pathologic protein deposits develop within specific brain regions independent of disease variant

Interestingly, cortical layer I had the least amount of significant protein differences when comparing disease groups to NHC, where only 4% of all significant proteins were exclusively expressed within cortical layer I (Fig. 2D). The white matter and cortical layers II-V of the cortex shared 26% of the total significant protein change. Additionally, 21% of all proteins were significantly different when comparing any disease group vs NHC in all three cortical subregions assessed (Fig. 2D). Collectively, these data suggest that individual cortical and subcortical layers may be more likely to reveal patterns of protein deposition than others.

### Amyloid and dopaminergic protein biomarkers transcend cortical subregion specificity across multiple variants of dementia

**Fig. 3:**
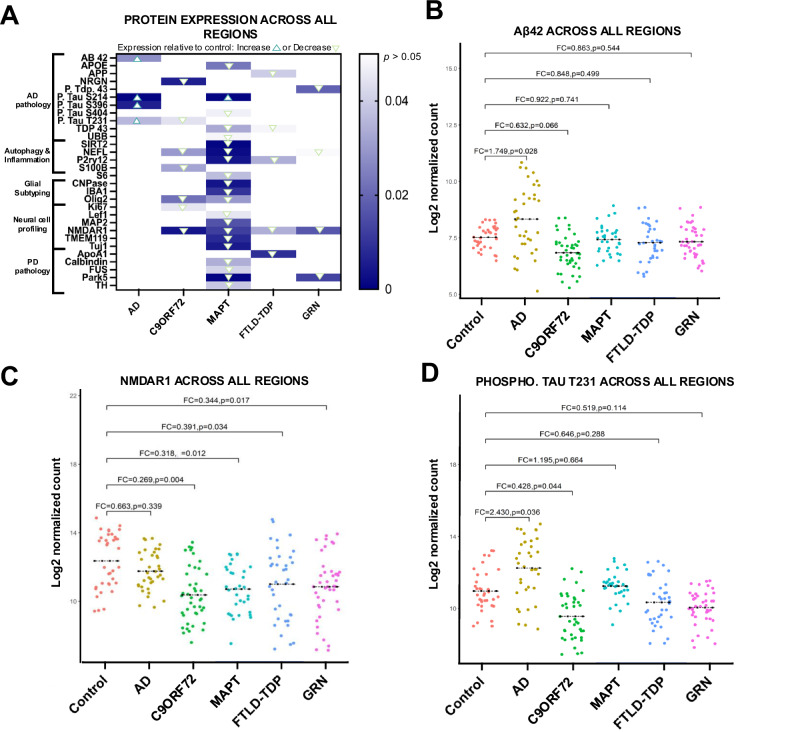
DSP identifies significant disease dependent protein expression changes irrespective of laminar cortical and subcortical layers of the brain. **A** Heatmap of differentially regulated proteins when comparing all regions of interest. Linear mixed model regression analysis was used to identify significantly upregulated proteins disease groups are indicated as any value above zero but less than *p* < 0.05. Significantly (*p* < 0.05) downregulated proteins are indicated by any value below zero. **B**–**D**Individual protein markers of interest independent of subregion specificity (all ROIs merged). Generalized linear regression comparing Log2 normalized gene expression via quantification of oligonucleotide barcode reads (counts) between disease groups was considered significant when *p* < 0.05. All counts were normalized to ROI area and internal controls.

The final analysis compared all ROIs (*n* = 4 per layer) collected across all cortical sublayers from every patient, where all disease ROIs were compared against each other and NHC (Fig. 3). As anticipated a handful of canonical biomarkers of neurodegenerative pathology, such as Aβ42, NMDAR1, and phosphorylated tau T231, revealed differential regulation as compared to NHC independent of ROI subregion (Fig. 3A–D; Supplementary Table 4). On the contrary, other popular canonical markers of neurodegeneration associated with dementia appear to be subregion-specific. For example, amyloid precursor protein (APP), a protein commonly associated with dementia, only indicated significant downregulation relative to NHC in cortical layer I and cortical layers II-V of the cortex but not the subcortical white matter (Fig. 2; Supplementary Tables 1–3). Of additional interest was tyrosine hydroxylase, an indicator of dopamine synthesis that is commonly decreased in Parkinsonism-associated dementia, was only found to significantly decrease from NHC in the internal sublayer of the cortex with no significant change in expression in any other investigated ROI (Fig. 3; Supplementary Table 4). These data suggest that not all protein regulation (observed from the chosen panel used within this study), is localized to a single layer. In the context of the 5 dementia variants investigated, some protein regulation may be domestic to each cortical layer, while other disease relevant proteins reveal a global response across the cortex.

## Discussion

FTLD is characterized by the progressive breakdown of both the anterior frontal and temporal lobes of the brain^1,2^ and is one of the most common causes of early onset dementia. Within the web of FTLD, there is vast heterogeneity in pathologic protein deposition in both the frontal and temporal cortices, resulting in a spectrum of clinical symptomology and pathology^13–18^. Therefore, the clinicopathologic diagnosis of FTLD and associated comorbidities can be extremely challenging^19^, and the pathogenic mechanisms underlying disease onset and progression continue to be elusive.

The cortex is highly specialized, with each layer serving a distinct role. It remains unclear as to what drives unique subregion dependent protein deposition due to disease. By leveraging the NanoString GeoMx^TM^ DSP technology we were able to demonstrate the extensive proteomic differences induced by disease and guided by cortical and subcortical laminar region. Not only did we further validate Aβ42 and phosphorylated tau regulation modulation in the cortex and subcortical white matter layer because of disease, but we more distinctly parsed which communities of protein biomarkers also changed in conjunction with these canonical pathologic indicators. We identified distinct communities of proteins that were unique to each cortical subregion, elucidating a critical role spatial proteomics can play in further characterizing the complex mechanisms of neurodegenerative disease.

In addition to further validation of canonical biomarkers of FTLD, we were able to distinguish phosphorylation patterns of pathological proteins (like tau) that were unique to each cortical subregion. These data reveal that phosphorylated tau protein deposits are likely unique to cortical and subcortical subregion. Although there is substantial evidence of various phosphorylated tau protein deposition in multiple regions of the brains of FTLD patients^20–22^, our data further solidify that phosphorylated tau may be subregion dependent within the human cortical and subcortical brain layers.

Of interest are the roles that inflammation and the immune system may play in subsequent brain region-specific protein deposition. P2ry12, a marker associated with broad microglia activation, significantly decreased in the subcortical white matter of only MAPT (also referred to as FTLD-tau) patients and also decreased within layers II-V of the cortex in AD, C9ORF72, MAPT, FTLD-TDP and GRN patients (Fig. 2). IBA1, another general marker for activated microglia, is significantly downregulated in MAPT patients within the white matter, layer I and layers II-V, as compared to NHC (Fig. 2). Depletion in IBA1 positive microglia in the human brain has been reported previously in association with both early- and late-onset AD^23–25^ however within our data set we only observe this phenotype in MAPT. Additionally, we identified a significant depletion in Sirt2 within the white matter, layer I and layers II-V, as compared to NHC (Fig. 2). Depletion of Sirt2 may be an indicator of mitochondrial stress and resulting apoptosis^26^. CNPase, a marker for myelin integrity and maintenance^27^, was significantly downregulated in MAPT patients as compared to NHC within the white matter, layer I and layers II–V, (Fig. 2). A decrease in p2ry12 and IBA1-positive microglia paired with dysfunctional myelin machinery and an increase in apoptotic features in MAPT patients may indicate dysregulated immune activity. These data suggest that MAPT induces the largest proteomic dysregulation in white matter and cortical layers I, II-V, as compared to what we detected in FTLD, AD, and NHC samples.

Although our data support distinct cortical subregion proteomic profiles, our analysis also indicates that a handful of mislocalized proteins transcend cortical layer specificity (Fig. 3). As expected, increased deposition of Aβ42 was evident in AD tissue when all regional ROIs were compared against NHC (Fig. 3). Interestingly NMDAR1 significantly decreased in C9ORF72, MAPT, FTLD-TDP and GRN tissue relative to NHC when all ROIs were combined (Fig. 3). NMDAR1 protein expression has been reported to decrease due to glutamatergic synaptic depression in tissue burdened with either tau^28^ or Aβ pathology^29^. Our data reveal that NMDAR1 may represent a viable biomarker for multiple variants of FTLD but not AD (Fig. 3).

Previous proteomic studies investigating FTLD or related dementia in human brains have assessed regional and/or cell-specific protein content^19,30,31^. NanoString DSP has been successfully used to delineate region-specific immune microenvironments associated with brain samples from individuals pathologically diagnosed with the TREM2 risk variant for AD^32^ and in separate study, parse neuroprotective protein regulation in brain samples from individuals resilient to AD cognitive consequence^33^. However, no study has reported multiplexed detection of different pathologic proteins of interest within each subregion of the cortex across multiple syndromes of FTLD. Common techniques studying protein-associated pathology such as mass spectrometry and western blotting are still highly relevant and used widely in the field. NanoString DSP does not replace these techniques but supplements these well-established tools by enabling preservation of the spatio-anatomical localization of proteins of interest. For example, in induced pluripotent stem cell (iPSC)-derived neurons from patients with various FTLD mutations, a dysregulated relationship between tau and mitochondrial proteins was identified using ascorbic acid peroxidase (APEX) combined with quantitative affinity purification mass spectrometry (AP-MS)^30^. Although the authors were able to provide insight on structural morphology by imaging the neurons, these data do not provide insight into the relevance of anatomical location within the complex and heterogenous microenvironment of the brain. Additional recent reports have provided region-specific insight into the early PD proteome, where 8 brain regions were spatially investigated using MS^e^ label free proteomics^31^. Although this method provides excellent region-specific protein interactions, the data set is specific to early PD and does not provide insight into subregion specific protein regulation.

Although our findings are novel and significant, the studies herein suffer from several limitations that should be noted. The number of cases per disease group was relatively small (*n* = 6), with such high variation within said small groups, we chose to treat each ROI within each subregion as its own representative data point. Future studies should consider the variation within each patient and use these data for determination of ROI-independent statistical assessment. These data help build a basis for region-specific proteomic assessment of FTLD and provide diagnostic insight into the region-specific discrepancies between each syndrome. Transcriptome information and single-cell resolution could further separate mechanistic differences (such as providing insight into specific cell-type ligand-receptor interactions and cell-type neighborhood interactomes) between various FTLD syndromes. Future work using single cell spatial omics technology, like 10X Genomics or NanoString CosMx SMI, would provide additional insight into this critical line of research.

NanoString GeoMx^TM^ is a novel method that leverages the ability to spatially multiplex protein biomarkers of interest. With the understanding that the catalyst of FTLD can be patient specific and extremely variable^34–37^, the current frontier of FTLD and neurodegeneration research has moved towards spatial proteomics approaches, where focused brain region-specific assessments now enable investigators to gain additional insight into the pathologic mechanisms underlying this spectrum of neurodegenerative syndromes. Our data provide a unique perspective on subregion-specific protein expression and/or depositions in various syndromes of FTLD that traditional proteomic approaches are unable to capture with precision. Specifically, the MAPT spatial proteome appears to be enriched with the largest immune- and inflammation-related dysregulation across both gray and white matter in the cortex. By spatially mapping protein expression within the cortex of five different variants of dementia we were able to catalog and continue to parse the brain region- and disease-specific proteome of various dementia syndromes. While a bit surprising to us, we find that although some traditional histopathologic markers of interest to dementia researchers selectively localize to specific brain regions of the cortex, other potentially pathologic protein deposits span wider anatomical areas of the brain. These data lay a foundation for additional work aimed at separating out common and shared mechanisms underlying various forms of FTLD disease in variant-specific future biomarker discovery efforts.

## Methods

### Cases

Postmortem tissue was de-identified and provided by the Department of Pathology at Emory University School of Medicine. All tissue collection and assessment were completed in accordance with Emory University and the University of Florida Institutional Review Board (Clinical Research in Neurology:Patient Registry CR2_IRB00024959 and IRB00024959).

Cortical tissue (Broadmann area 9) was obtained from Emory University Goizueta Alzheimer’s Disease Research Center (ADRC) brain bank on 30 patients diagnosed with neurological disease and 6 neurologically healthy controls (Table 1; Supplementary Table 5). The five neurological diseases assessed include Alzheimer’s disease (AD), amyotrophic lateral sclerosis associated with a C9 expansion mutation (C9ORF72), frontotemporal lobar degeneration with parkinsonism-17 (MAPT), frontotemporal lobar degeneration with TPD-43-immunoreactive pathology (FTLD-TDP), and progranulin (GRN) mutation related FTLD-GRN. Disease and neurologically healthy control groups were matched as closely as possible for age and sex (Table 1; Supplementary Table 5). Neuropathologic diagnosis and characterization of disease associated pathology were conducted at Emory University (MGT) following standardized criteria^38–40^. Due to the low case number in each group (*n* = 6) and such high variation within said small groups, we chose to treat each ROI within each subregion as its own representative data point. Individual case Braak staging and secondary neuropathology are detailed in Supplementary Table 5.

**Table 1:** Case demographics, cognitive status and neuropathologic outcomes

	AD *N* = 6	C9orf72 *N* = 6	MAPT (FTLD-Tau) *N* = 6	FTLD-TDP *N* = 6	FTLD-GRN *N* = 6	NHC *N* = 6
Sex, Female/Male	3/3	3/3	4/2	3/3	3/3	2/4
Average age at death, years Average $\pm$ SD Median (IQR)	63.3 $\pm$ 4.8 63 (3.5)	66 $\pm$ 4.8 66.5 (3.25)	58.3 $\pm$ 9.7 62 (7.75)	63.7 $\pm$ 4.6 62.5 (6)	63.3 $\pm$ 3.8 62 (1.5)	63.3 $\pm$ 3.9 62.5 (3.75)
Disease duration, years Average $\pm$ SD Median (IQR)	9.6 $\pm$ 3.4 9 (4.25)	7.9 $\pm$ 4.5 9 (4.5)	6.1 $\pm$ 2 5 (4.5) 1 case not reported	5.5 $\pm$ 3.3 6.5 (4.75)	6.2 $\pm$ 2.0 6 (1.125)	n.a.
Reported race/ethnicity	5 white 1 black	6 white	6 white	6 white	1 other 5 white	2 white 4 black
ABC Score Average $\pm$ SD Median (IQR)	3 $\pm$ 0 3 (0)	0.6 $\pm$ 0.5 1 (1) 1 case not reported	0 0 (0) 3 cases not reported	0.7 $\pm$ 0.8 0.5 (1)	0.7 $\pm$ 0.5 1 (0.75)	1 $\pm$ 1 1 (0.75)
CERAD Score Average $\pm$ SD Median (IQR)	3 $\pm$ 0 3 (0)	0 0 (0)	0.7 $\pm$ 1 0 (1.5)	0.5 $\pm$ 1 0 (0)	0 0 (0)	0.5 $\pm$ 1 0 (0)
ThaI Amyloid Score Average $\pm$ SD Median (IQR)	5 $\pm$ 0 5 (0)	0.8 $\pm$ 0.8 1 (0.75)	1 $\pm$ 2 0.5 (2.5)	1 $\pm$ 2 0.5 (1)	1 $\pm$ 1 1.5 (1.75)	2$\,\pm$ 2 1.5 (1.75)

ABC (Amyloid/Braak/CERAD) Score: score reflecting the degree of AD neuropathologic change (0 = none, 1 = low, 2 = intermediate, 3 = high)^38^.

CERAD Score: Consortium to Establish a Registry for Alzheimer’s Disease score reflecting the frequency of neuritic plaques in the neocortex (0 = none, 1 = sparse, 2 = moderate, 3 = frequent)^44^.

ThaI Amyloid Score: score reflecting the distribution of immunopositive amyloid deposits throughout the brain ranges from 0=no deposits through 5=deposits throughout the brain, including the cerebellum)^45^.

*AD* Alzheimer’s disease, *C9orf72* chromosome 9 open reading frame 72, *MAPT* microtubule associated protein tau, *FTLD* frontotemporal lobar degeneration, *TDP* *=* *TAR* DNA-binding protein 43, *GRN* granulin precursor, *NHC* neurologically healthy control, *IQR* Interquartile range.

### NanoString GeoMx^TM^ digital spatial profiling (DSP)

Paraformaldehyde-fixed prefrontal cortex (Brodmann area 9) samples were sectioned at 5 µm. Sections were incubated with the NanoString GeoMx^TM^ human neuroscience protein panel (Fig. 1C); which includes 75 UV-cleavable protein probes and 3 antibody visualization markers against nuclear DNA (Syto13), microglia (IBA1, Cat#MABN92-AF647) and astrocytes (GFAP, Cat#8152S) (Fig. 1A and visualization in Fig. 1B). Tissues were baked at 37 °C overnight and then rehydrated according to the manufacturer’s instructions (MAN-10089-10). Antigen retrieval was performed using freshly prepared Citrate Buffer of pH 6 in a pressure cooker for 15 min, and then incubated with the human neuroscience panel overnight at 4 °C following the manufacturer’s instructions (MAN-10089-10). Visualization markers listed above were used to generate 20X florescent images scanned by GeoMx^TM^ DSP. Regions of interest (ROIs) were chosen to assess established neuropathological hallmarks in defined areas associated with neurodegeneration and pathology. Specifically, we assessed the following three ROIs: The molecular layer (cortical layer 1), internal layer (cortical layers II-V), and the white matter. Cortical layer ROIs were differentiated by 1) density of nuclear staining and geographic location relative to tissue edge, and 2) validated by observing known location specific protein pathology. For example, in Alzheimer’s disease, amyloid-β plaques are observed throughout all cortical layers whereas tau pathology associated with neurofibrillary tangles (NFTs) is observe in layers III and V^41^. Similarly, TDP-43 pathology in FTLD-TDP (including C9ORF72 and GRN) is observed in layers II-V^42^. Neuronal loss in cortical layers is a common finding across several dementia related neurodegenerative diseases; thus, we designed our “internal layer” ROI to encompass layers II-V to increase signal-to-noise and ensure we captured enough signal to detect neuropathology’s. ROIs were drawn in circles, and size of the ROI was based on capturing the maximum area of the given region. Twelve ROIs per case were chosen, where four ROIs were drawn per cortical layer (Fig. 1B). Once ROIs were selected on the GeoMx^TM^ digital spatial profiler, UV light was used to illuminate the ROIs—cleaving the protein specific UV-cleavable oligonucleotide tags and transferred to 96-well plates. ROI specific cleaved tags then hybridized overnight with probe R which includes unique to six-color optical barcodes. Optical barcodes were then quantified on the NanoString nCounter MAX/FLEX by counting the number of barcodes specific to each protein present within each ROI. The anonymized data supporting the findings within this manuscript may be accessed via Zenodo online data repository URL: 10.5281/zenodo.14143622.

### GeoMx^TM^ DSP analysis

Raw protein expression data were input into NanoString DSPAPP V6.0 Analysis Software. The NanoString human neuroscience protein panel contains housekeeping controls GAPDH, S6 and H3; however, these internal controls can change in expression in the context of neurodegenerative disease^43^ and were thus not used to quality check (QC) raw protein counts. Therefore, positive normalization was preformed using external RNA controls consortium (ERCC), which acts as a QC for hybridization efficiency and technical variation. Negative internal controls included IgGs (Rb IgG, Ms IgG1, Ms IgG2a), which served as an additional QC measure that adjusts for any nonspecific target adherence of the probes. ROIs within each cortical layer of each case were then compared to other cortical layers across disease type, and when comparing across ROIs, total protein expression was normalized relative to surface area (geometric mean) of each ROI. Differential expression analysis was completed via a linear mixed model, adjusted for age and sex. *P*-values were adjusted for multiple analyses via Benjamini-Hochberg procedure. Heatmaps displaying *p*-values were created by exporting the normalized .csv File from GeoMx^TM^ analysis suite and importing into GraphPad Prism (Version 10.1.1).

## Supplementary information

Supplementary Tables

## Data Availability

All analyzed data sets within this study have been provided in the primary figures or supplementary material. All anonymized data files are publicly available via Zenodo online public data respository at: 10.5281/zenodo.14143622.

## Abreviations

AD: Alzheimer’s disease
Aβ: Amyloid-β
ABC score: Activities of daily Living, Behavioral and psychological Symptoms of Dementia, and Cognitive Function
AP-MS: quantitative affinity purification mass spectrometry
APP: Amyloid precursor protein
APEX: Ascorbic acid peroxidase
ApoA1: Apolipoprotein A1
ALS: Amyotrophic lateral sclerosis
bvFTD: Behavioral variant frontotemporal dementia
CERAD score: Consortium to Establish a Registry for Alzheimer’s Disease
C9ORF72: chromosome 9 open reading frame 72
DSP: Digital spatial profiling
FDR: False discovery rate
FTD: Frontotemporal dementia
FTLD: Frontotemporal lobar degeneration
GAPDH: glyceraldehyde-3-phosphate dehydrogenase
GFAP: Glial fibrillary acidic protein
GRN: progranulin mutation related frontotemporal lobar degeneration
IBA1: Ionized calcium binding adaptor molecule 1
IPSC: Induced pluripotent stem cell
IQR: Interquartile range
Lef1: Lymphoid Enhancer Binding Factor 1
MAPT: microtubule associated protein tau
Map2: Microtubule associated protein 2
NEFL: Neurofilament light
NHC: Neurologically healthy control
NMDAR1: Glutamate [NMDA] receptor subunit 1
Park5 (UCH-L1): Ubiquitin carboxyl-terminal hydrolase isozyme L1
PD: Parkinson’s disease
PGRN: Progranulin
P2ry12: Purinergic receptor P2Y12
P53: Tumor protein 53
ROI: Region of interest
S6: Ribosomal protein S6
TARDBP: Transactive DNA-binding protein gene
TDP-43: Transactive response DNA binding protein of 43 kDa
UBB: Ubiquitin
