# Supplementary material for: Multiplex digital spatial profiling identifies subregion dependent targeted proteome changes across variants of dementia
Source: NPJ Dement. 2025 Jun 3;1(1):10. doi: 10.1038/s44400-025-00010-6 (PMC12133586; doi:10.1038/s44400-025-00010-6)
Supplement: Supplementary file 1 — Supplementary Tables [file 44400_2025_10_MOESM1_ESM.pdf]

Supplementary Table 1

Individual protein count significance and fold change in the white matter of the cortex and compared to NHC. NS represents no significant change when compared to NHC. An up arrow indicates a positive fold change and a down arrow indicates a negative fold change when comparing the disease group to NHC.

	AD	C9ORF72	MAPT	FTLD-TDP	GRN
AB 42	NS	↓ p = 0.011, FC = 0.557	NS	NS	NS
ApoA1	NS	↓ p = 0.025, FC = 0.363	↓ p = 0.001, FC = 0.209	NS	NS
BDNF	NS	NS	↑ p = 0.011, FC = 2.147	NS	NS
Calbindin	NS	NS	↓ p = 0.028, FC = 0.351	NS	NS
CD11b	NS	NS	↓ p = 0.022, FC = 0.463	NS	NS
CNPase	NS	NS	↓ p = 0.002, FC = 0.124	NS	NS
FoxA2	↓ p = 0.032, FC = 0.488	NS	NS	NS	NS
FUS	NS	NS	↓ p = 0.036, FC = 0.511	NS	NS
GAPDH	NS	↓ p = 0.034, FC= 0.366	NS	NS	NS
IBA1	NS	NS	↓ p = 0.017, FC = 0.285	NS	NS
Lef1	↓ p =0.014, FC= 0.562	NS	↓ p = 0.019, FC = 0.579	NS	NS
MAP2	NS	NS	↓ p = 0.034, FC = 0.189	↓ p =0.020, FC = 0.159	NS
Ms.IgG2a	↓ p =0.009, FC= 0.534	NS	NS	NS	NS
NEFL	NS	↓ p = 0.021, FC = 0.409	NS	NS	NS
NMDAR1	NS	NS	↓ p = 0.029, FC= 0.353	NS	NS
NRGN	NS	↓ p = 0.006, FC = 0.203	NS	NS	NS
Olig2	NS	NS	↓ p = 0.027, FC = 0.432	NS	NS
P2ry12	NS	NS	↓ p = 0.005, FC = 0.210	NS	NS
p53	↓ p = 0.048, FC =0.626	NS	↓ p = 0.016, FC = 0.567	NS	NS
Park5	NS	↓ p = 0.026, FC= 0.362	↓ p <0.001, FC.= 0.154	NS	↓ p = 0.009, FC – 0.298
polyGP	↓ p = 0.017, FC = 0.561	↓ p = 0.009, FC = 0.553	↓ p = 0.043, FC = 0.636	NS	NS
PSEN1	NS	NS	NS	↓ p = 0.034, FC = 0.457	NS
Phospho. Tau S214	NS	NS	↓ p = 0.003, FC = 5.946	NS	↓ p = 0.043, FC = 3.206
Phospho. Tau S404	↓ p = 0.046, FC = 0.422	NS	↓ p = 0.009, FC = 0.313	NS	NS
Phospho. Tau T231	NS	↓ p = 0.042, FC = 0.441	NS	NS	NS
SIRT2	NS	NS	↓ p = 0.006, FC = 0.344	NS	NS
SNCA filament	NS	NS	↓ p = 0.036, FC = 0.385	NS	NS
Tau	NS	NS	↓ p = 0.009, FC= 0.263	NS	NS
TDP43	NS	NS	↓ p = 0.032, FC = 0.546	NS	NS
TMEM119	NS	NS	↓ p = 0.004, FC = 0.151	NS	NS
Tuj1	NS	NS	↓ p = 0.002, FC = 0.334	NS	NS
UBB	NS	NS	↓ p = 0.008. FC = 0.257	NS	↓ p = 0.043, FC = 0.362

Supplementary Table 2

Individual protein count significance and fold change in cortical layer II-V of the compared to NHC. NS represents no significant change when compared to NHC. An up arrow indicates a positive fold change and a down arrow indicates a negative fold change when comparing the disease group to NHC.

	AD	C9ORF72	MAPT	FTLD-TDP	GRN
AB 42	↑ p = 0.003, FC = 2.566	NS	NS	NS	NS
APP	NS	NS	↓ p = 0.024, FC = 0.294	↓ p = 0.016, FC = 0.0271	NS
BACE1	NS	NS	↓ p = 0.033, FC = 0.325	NS	NS
Calbindin	NS	NS	↓ p = 0.021, FC = 0.295	NS	NS
CNPase	NS	NS	↓ p = 0.019, FC = 0.279	↓ p = 0.050, FC = 0.348	NS
FUS	NS	NS	↓ p = 0.020, FC – 0.401	NS	NS
GAPDH	NS	NS	↓ p = 0.004, FC = 0.386	NS	NS
GPNMB	NS	NS	↓ p = 0.036, FC = 0.469	NS	NS
HISTONE H3	NS	NS	↓ p = 0.037. FC = 0.216	NS	NS
IBA1	NS	NS	↓. p = 0.007, FC 0.222	NS	NS
IQGAP2	NS	NS	↓ p = 0.023, FC = 0.469	NS	NS
Ki67	NS	NS	NS	↓ p=0.045, FC = 0.440	NS
MAP2	NS	NS	NS	NS	NS
NEFL	NS	↓ p = 0.041, FC = 0.413	NS	NS	↓ p = 0.039, FC = 0.409
NMDAR1	NS	NS	NS	↓ p = 0..047, FC = 0.301	NS
NRGN	NS	↓ p = 0.026, FC = 0.296	NS	NS	NS
Olig2	NS	NS	↓ p = 0.018, FC = 0.382	↓ p = 0.032, FC = 0.420	
P2ry12	↓ p = 0.015, FC = 0.402	↓ p = 0.010, FC = 0.379	↓ p =0.002, FC = 0.291	↓ p = 0.012, FC =0.389	↓ p = 0.012, FC = 0.390
p53	NS	NS	↓ p = 0.040, FC = 0.475	NS	
Park5	NS	NS	↓ p <0.001, FC = 0.191	NS	↓ p = 0.019, FC = 0.340
Phospho. Tdp. 43	NS	NS	NS	NS	↓ p = 0.016, FC = 4.004
PINK1	NS	↓ p= 0.044, FC 0.440	NS	NS	NS
Phospho. Tau S214	↑ p <0.001, FC = 12.596	NS	↑ p <0.001, FC = 4.949	NS	NS
Phospho. Tau S396	↑ p <0.001, FC = 11.277	NS	NS	NS	NS
Phospho. Tau 231	↑ p = 0.004, FC = 4.467	NS	NS	NS	NS
S6	NS	NS	↓ p = 0.008, FC = 0.238	↓ p = 0.046 FC = 0.351	NS
SIRT2	NS	NS	↓ p = 0.011, FC = 0.346	NS	NS
Tau	NS	NS	↓ p =0.044, FC = 0.348	NS	NS
TDP 43	NS	NS	↓ p = 0.010. FC = 0.408	↓ p=0.016, FC =0.436	NS
TH	NS	NS	↓ p.= 0.024, FC = 0.459	NS	NS
TMEM119	NS	NS	↓ p = 0.006, FC = 0.208	NS	NS
Tuj1	NS	NS	↓ p =0.020, FC = 0.451	NS	NS
UBB	NS	NS	NS	↓ p = 0.036, FC = 0.340	NS
vGlut1	NS	NS	NS	↓ p = 0.042, FC = 0.322	NS

Supplementary Table 3

Individual protein count significance and fold change in cortical layer I compared to NHC. NS represents no significant change when compared to NHC. An up arrow indicates a positive fold change and a down arrow indicates a negative fold change when comparing the disease group to NHC.

	AD	C9ORF72	MAPT	FTLD-TDP	GRN
AB 42	↑ p = 0.002, FC = 3.261	NS	NS	NS	NS
APP	NS	NS	NS	↓ p = 0.038, FC = 0.285	NS
CNPase	NS	NS	↓ p = 0.026, FC = 0.238	NS	NS
GAPDH	NS	↓ p = 0.036, FC = 0.400	NS	NS	NS
IBA1	NS	NS	↓ p = 0.021, FC = 0.316	NS	NS
NMDAR1	NS	↓ p = 0.007, FC = 0.160	↓ **p = 0.039, FC = 0.236**	↓ p = 0.047, FC = 0.236	↓ p = 0.025, FC = 0.221
NRGN	NS	↓ p= 0.018, FC = 0.281	NS	NS	NS
Park5	NS	NS	↓ p = 0.025, FC = 0.418	NS	NS
Phospho. Tdp. 43	NS	↑ p= 0.026, FC = 4.511	NS	NS	↑ p = 0.029, FC = 4.387
Phospho. Tau S214	↑ p <0.001, FC = 11.825	NS	↑ p = 0.003, FC – 4.650	NS	NS
Phospho. Tau S396	↑ p < 0.001, FC =13.871	NS	↑ p = 0.075, FC = 2.905	NS	NS
Phospho. Tau T231	↑ p < 0.001, FC = 6.426	NS	NS	NS	NS
S100B	NS	↓ p = 0.045, FC = 0.494	NS	NS	NS
SIRT2	NS	NS	↓ p = 0.002, FC 0.111	NS	↓ p = 0.039, FC = 0.270
Tuj1	NS	NS	NS	NS	NS

Supplementary Table 4

Individual protein count significance and fold change when all ROIs are combined and compared to NHC. NS represents no significant change when compared to NHC. An up arrow indicates a positive fold change and a down arrow indicates a negative fold change when comparing the disease group to NHC.

	AD	C9ORF72	MAPT	FTLD-TDP	GRN
AB 42	↑ p = 0.003, FC = 2.566	NS	NS	NS	NS
APP	NS	NS	↓ p = 0.024, FC = 0.294	↓ p = 0.016, FC = 0.0271	NS
BACE1	NS	NS	↓ p = 0.033, FC = 0.325	NS	NS
Calbindin	NS	NS	↓ p = 0.021, FC = 0.295	NS	NS
CNPase	NS	NS	↓ p = 0.019, FC = 0.279	↓ p = 0.050, FC = 0.348	NS
FUS	NS	NS	↓ p = 0.020, FC – 0.401	NS	NS
GAPDH	NS	NS	↓ p = 0.004, FC = 0.386	NS	NS
GPNMB	NS	NS	↓ p = 0.036, FC = 0.469	NS	NS
HISTONE H3	NS	NS	↓ p = 0.037. FC = 0.216	NS	NS
IBA1	NS	NS	↓. p = 0.007, FC 0.222	NS	NS
IQGAP2	NS	NS	↓ p = 0.023, FC = 0.469	NS	NS
Ki67	NS	NS	NS	↓ p=0.045, FC = 0.440	NS
MAP2	NS	NS	NS	NS	NS
NEFL	NS	↓ p = 0.041, FC = 0.413	NS	NS	↓ p = 0.039, FC = 0.409
NMDAR1	NS	NS	NS	↓ p = 0..047, FC = 0.301	NS
NRGN	NS	↓ p = 0.026, FC = 0.296	NS	NS	NS
Olig2	NS	NS	↓ p = 0.018, FC = 0.382	↓ p = 0.032, FC = 0.420	
P2ry12	↓ p = 0.015, FC = 0.402	↓ p = 0.010, FC = 0.379	↓ p =0.002, FC = 0.291	↓ p = 0.012, FC =0.389	↓ p = 0.012, FC = 0.390
p53	NS	NS	↓ p = 0.040, FC = 0.475	NS	
Park5	NS	NS	↓ p <0.001, FC = 0.191	NS	↓ p = 0.019, FC = 0.340
Phospho. Tdp. 43	NS	NS	NS	NS	↓ p = 0.016, FC = 4.004
PINK1	NS	↓ p= 0.044, FC 0.440	NS	NS	NS
Phospho. Tau S214	↑ p <0.001, FC = 12.596	NS	↑ p <0.001, FC = 4.949	NS	NS
Phospho. Tau S396	↑ p <0.001, FC = 11.277	NS	NS	NS	NS
Phospho. Tau 231	↑ p = 0.004, FC = 4.467	NS	NS	NS	NS
S6	NS	NS	↓ p = 0.008, FC = 0.238	↓ p = 0.046 FC = 0.351	NS
SIRT2	NS	NS	↓ p = 0.011, FC = 0.346	NS	NS
Tau	NS	NS	↓ p =0.044, FC = 0.348	NS	NS
TDP 43	NS	NS	↓ p = 0.010. FC = 0.408	↓ p=0.016, FC =0.436	NS
TH	NS	NS	↓ p.= 0.024, FC = 0.459	NS	NS
TMEM119	NS	NS	↓ p = 0.006, FC = 0.208	NS	NS
Tuj1	NS	NS	↓ p =0.020, FC = 0.451	NS	NS
UBB	NS	NS	NS	↓ p = 0.036, FC = 0.340	NS
vGlut1	NS	NS	NS	↓ p = 0.042, FC = 0.322	NS

Supplementary Table 5 . Individual case demographics and cognitive status.

Case	Disease	Primary Neuropathologic Diagnosis	Other Neuropathologic Diagnosis - AD	Other Neuropathologic Diagnosis - LBD	Other Neuropathologic Diagnosis - TDP	Other Neuropathologic Diagnosis - tau	Braak Stage	ABC	CERAD	Thal	Age at Onset	Age at Death	Duration (years)	ApoE	Race	Sex	Clin Dx	Family Hx
					AD		C9ORF72				MAPT			FTLD-TDP			GRN	
1	FTLD-TDP	FTLD-TDP					[illegible]	[illegible]	[illegible]	[illegible]	[illegible]	[illegible]	[illegible]	[illegible]	[illegible]	[illegible]	[illegible] S	[illegible]
2	FTLD-TDP	FTLD-TDP					I	1	0	1	56	64	8	E3/4	w	f	FTD	Yes
3	AD	AD		LBD-amygdala			VI	3	3	5	56	64	8	E3/4	w	m	AD	
4	AD	AD		LBD-amygdala			VI	3	3	5	59	72	14	E3/4	w	f	AD	Yes
5	Control	AD		LBD-amygdala			VI	3	3	5	56	64	8	E3/4	w	m	AD	
6	AD	AD		LBD-amygdala	LATE-NC (stage 1)		VI	3	3	5	51	64	13	E3/4	w	f	AD	na
7	GRN	FTLD-TDP (GRN mutation)					0	0	0	0	52	62	10	E3/3	other	f	FTD	Yes
8	FTLD-TDP	FTLD-TDP					II	0	0	0	62	71	9	E3/3	w	f	FTD	No
9	AD	AD			LATE-NC (stage 1)		V	3	3	5	52	62	10	E3/4	w	m	AD	No
10	Control	Control					I	0	0	0	na	59		E2/3	b	m	ischemic heart disease and fibrosis	
11	AD	AD			LATE-NC (stage 2)		VI	3	3	5	52	60	8	E4/4	b	m	Pick's disease	Yes
12	CD9-ORF72	FTLD-TDP (C9 expansion)					III	1	0	2	57	66	9	E3/3	w	m	FTD/Pick's disease	Yes
13	GRN	FTLD-TDP (GRN mutation)			LATE-NC (stage 3)		I	1	0	2	67	71	4.5	E3/4	w	m	AD	Yes
14	AD	AD					VI	3	3	5	53	58	5	E3/4	w	f	AD	No
15	FTLD-TDP	FTLD-TDP	AD				IV	2	3	5	58	60	2	E3/4	w	f	FTD	Yes
16	MAPT	FTDP-17 (P301L)					na	0	0	0	56	60	4	E3/3	w	f	FTDP-17 (P301L)	Yes
17	MAPT	FTDP-17 (P301L)					0	0	0	0	56	64	8.5	E3/4	w	m	FTDP-17 (P301L)	Yes
18	FTLD-TDP	FTLD-TDP					II	1	0	1	66	67	1	E2/3	w	m	FTD; CBD vs. prion disease	Yes
19	GRN	FTLD-TDP (GRN mutation)		LBD-amygdala			I	1	0	2	58	62	4.5	E3/4	w	m	CBD	Yes
20	GRN	FTLD-TDP (GRN mutation)					I	0	0	0	57	63	6	E2/3	w	m	primary progressive aphasia	No
21	CD9-ORF72	FTLD-TDP (C9 expansion)			ALS (C9 expansion)		0	0	0	0	55	57	1.5	na	w	f	ALS+FTD	na
22	CD9-ORF72	FTLD-TDP (C9 expansion)		LBD-amygdala			IV	1	0	1	60	70	10	E2/3	w	f	FTD-primary progressive aphasia; PSP	No
23	GRN	FTLD-TDP (GRN mutation)					III	1	0	1	55	61	6	E3/3	w	f	FTD	Yes
24	Control	Control					II	1	0	1	na	61		na	b	f	diabetes; hx hip replacement	
25	CD9-ORF72	FTLD-TDP (C9 expansion)			ALS		II	0	0	0	62	66	4	E4/4	w	m	FTD; ALS	No
26	CD9-ORF72	FTLD-TDP (C9 expansion)			ALS	Tau pathology-mild	na	na	0	1	58	67	9		w	m	FTD; ALS	Yes
27	MAPT	FTDP-17 (P301L)					na	0	0	0	51	56	5	na	w	f	FTD (probable FTDP-17)	Yes
28	CD9-ORF72	FTLD-TDP (C9 expansion)			TDP-43 incl in sp cord neurons		III	1	0	1	56	7	14	E3/3	w	f	FTD	Yes
29	GRN	FTLD-TDP (GRN mutation)					I	1	0	2	55	61	6	E3/4	w	f	FTD (GRN mutation)	Yes
30	Control	Control					I	1	0	2	na	70		E3/3	b	m	control	Yes
31	MAPT	FTDP-17 (P301L presumed)	AD-probable				na	na	2	4	56	65	9	E2/3	w	f	FTD	Yes
32	MAPT	FTDP-17 (G389R)					na	na	0	1	36	40	4	E3/3	w	m	FTD	Yes
33	Control	Control					I	0	0	0	na	65	na	E3/3	w	f	systemic primary amyloidosis, AL type	na
34	Control	Control					II	1	0	2	na	61		E3/4	b	m	diabetes, renal disease, congestive heart failure	
35	FTLD-TDP	FTLD-TDP		LBD-amygdala			0	0	0	0	52	59	7	E3/3	w	m	FTD	Yes
36	MAPT	FTDP-17 (R406W)	AD-definite				na	na	2	3	na	65	na	na	w	f	FTDP-17 (R406W)	Yes
